# Remarks on the Management of Suppuration, Complicating Tuberculous Disease of the Bones and Joints1Read before the Medical Society of the State of New York, February 8, 1893.

**Published:** 1893-05

**Authors:** Louis A. Weigel

**Affiliations:** Rochester, N. Y., Assistant Orthopedic Surgeon to the Rochester City Hospital; 43 Mortimer Street


### REMARKS ON THE MANAGEMENT OF SUPPURATION, COMPLICATING TUBERCULOUS DISEASE OF THE BONES AND JOINTS.[1]

By LOUIS A. WEIGEL, M. D, Rochester, N. Y.,

Assistant Orthopedic Surgeon to the Rochester City Hospital.

With the advanced knowledge of surgical pathology, many points in the etiology of tuberculous bone disease have been cleared up, and the general methods of treatment are supposed to be more scientific and accurate. An examination, however, of the literature on the treatment of tuberculous bone abscesses clearly proves that surgeons are far from being agreed as to the proper course to pursue in any given case. Fifty years ago, Sir Benjamin Brodie advised amputation in cases of extensive or protracted suppuration as the best method of stopping the progress of the bone disease which gives rise to it, and save the patient's life. It is not surprising that, from this extremely radical procedure, surgeons drifted to the other extreme of ultra-conservatism and advised against all operative interference in tuberculous bone disease, save under exceptional conditions. While the advent of antiseptic surgery has modified former opinions with reference to the justifiability of operative interference in the class of cases under discussion, the advisability of aspiration, incision, or drainage, etc., is not generally admitted. Unanimity of opinion may be out of the question; at the same time it ought not to be difficult to establish some well-defined principles in the treatment of tuberculous abscesses.

The days of expectancy in the management of serious diseases of the bones and joints and their complications are past. While it is sometimes admissible or even advisable to treat abscesses expectantly, there can be no reasonable objection to operative

1. Read before the Medical Society of the State of New York, February 8, 1893.

interference in a large proportion of cases. Judson's[1] statement that "the great number of scarred but healthy cripples who have recovered without surgical attention excludes the thought of malignancy, and even indicates that in every uncomplicated case a point will be reached when the destructive is replaced by a reparative process," can hardly be regarded as an argument in favor of non-interference. For it has been demonstrated by careful observation, that patients having the advantage of intelligent supervision and treatment usually recover more rapidly and with functionally better joints, and are less likely to develop abscesses during the progress of tuberculous bone disease. These results can fairly be attributed to the modern conservative method of treatment, which does not exclude the use of the knife. Conservatism must not be confounded with expectancy.

The element of time is of some importance in all tuberculous diseases. Prolonged suppuration from bone disease must necessarily have a deleterious influence upon the general health of a tuberculous subject. If, by any procedure, the natural duration of the disease can be shortened, it is a distinct gain to the patient. I am not prepared to admit that a child's time is of no value, so that it is a matter of indifference how protracted the duration of treatment may be in any given case. Simple protection and fixation of a joint is often sufficient to secure a good result; still, not infrequently, surgical interference may be necessary to avert extensive destruction of bone and prevent joint implication.

It may be true that in hospitals and among the poor, cases must be treated on a somewhat different plan than those occurring among the better classes. In the former, operations are frequently performed for the purpose of shortening the duration of treatment. Now, if, by such operative interference, good results are obtained in a comparatively shorter time, I see no reason why a similar method of treatment is not applicable in private practice.

It is a well-recognized fact that when a tuberculous focus is located near an articular surface, there is great danger of an extension of the disease into the joint. For this reason protection and functional rest are necessary in order to limit the extent of the tubercular ostitis, in its early stages. In some of the more accessible joints of the body, a focus can usually be located with some degree of accuracy. When this is possible, it is a comparatively simple matter to remove it absolutely by an operation and thus

1. *Medical Record*, May 18, 1889.

prevent joint implication, and diminish the probability of general infection. This method of treatment is especially applicable to disease about the ankle-joint. It should be remembered that softening, caseation, or suppuration, may take place in the bone before there are any marked external evidences of the destructive process going on within. An early diagnosis and appropriate treatment are the best safeguards against joint implication.

The two following cases, dissimilar in character, illustrate this point :

Case I. F. Z., *æt.* 5 years, examined in September, 1890, gave a history of a traumatism. His present symptoms consisted of slight pain in the ankle, and limping. Rest was advised and the parents requested to present the boy for re-examination four weeks later. The patient, however, was not seen again until January 29, 1891, when he was admitted to the Rochester City Hospital. At this time the joint was hot, swollen, and very painful, and presented the general appearance of an acute synovitis. I satisfied myself. however, that it was not a case of synovitis, but one of bone suppuration. As there was no improvement under treatment by rest, an operation was performed March 2, 1891. On exposing the internal malleolus, the bone was found to be of a bluish color and in an osteo-porotic condition. It was a simple matter to open the cavity in the bone and remove the pus and degenerated tissue found there, with the sharp spoon. An incision over the external malleolus revealed a similar condition, and was accordingly operated upon in the same way. The cavities were packed with iodoform gauze, and an antiseptic dressing applied. In less than three months the boy was discharged absolutely recovered. The general health of the patient began to improve within a week after the operation, and is now remarkably good. Within the past week I examined the boy again, and found an absolutely normal joint and no evidence of bone disease ; the leg, however, is one-half an inch shorter than its fellow.

Case II. F. L., *æt.* 8 years, an orphan asylum subject, was admitted to the hospital May 1, 1891. No previous history obtainable. Present symptoms consist of swelling over the internal malleolus, limping, and extreme tenderness on pressure. On May 25, 1891, the epiphysis was opened, a quantity of soft, cheesy matter removed, and the bone thoroughly curetted. After-treatment the same as in Case I. The patient made a rapid recovery, and was discharged from the hospital July 10, 1891. A recent examination of this patient demonstrates the permanency of the recovery. He is in excellent health, is free from pain, and has a normal joint.

In the first case the necessity of an operation could not be questioned, and the ultimate result abundantly proved its advisability. Whether the shortening is the direct result of the operation, or due to a tropho-neurotic lesion, is an open question. Atrophy of bone and muscle usually occurs in these cases, under all forms of treatment. In any event, the shortening is of less importance than a useful joint.

In the second case, recovery might have taken place without operative interference, by rest and fixation, continued for an indefinite period. The operation brought about a rapid recovery and removed all probability of secondary joint infection.

It may be of interest to add that an older brother of this patient died from spondylitis and general tuberculosis, about two months ago.

43 Mortimer Street.

